# Nondestructive Nonlinear Optical Microscopy Revealed the Blackening Mechanism of Ancient Chinese Jades

**DOI:** 10.34133/research.0266

**Published:** 2023-11-14

**Authors:** Yaxin Chen, Rong Wang, Minbiao Ji

**Affiliations:** ^1^ State Key Laboratory of Surface Physics and Department of Physics, Human Phenome Institute, Academy for Engineering and Technology, Key Laboratory of Micro and Nano Photonic Structures (Ministry of Education), Yiwu Research Institute of Fudan University, Fudan University, Shanghai 200433, China.; ^2^ Department of Cultural Heritage and Museology, Fudan University, Shanghai, China.

## Abstract

Jade is most valued in Chinese culture since ancient times. For unearthed jade artifacts, the alteration color resulting from weathering effects and human activities provides information for cultural heritage conservation, archaeology, and history. Currently, the noninvasive 3-dimensional characterization of jade artifacts with high chemical and spatial resolution remains challenging. In this work, we applied femtosecond pump–probe microscopy and second harmonic generation microscopy techniques to study the black alteration of an ancient jade artifact of the late Spring and Autumn period (546 to 476 BC). The direct cause of the “mercury alteration” phenomena was discovered to be the conversion of metacinnabar from buried cinnabar in the tomb. Furthermore, a 3-dimensional optical reconstruction of the black alteration was achieved, providing a high-resolution method for analyzing the blackening mechanism without the need of sample damage. Our approach opens up new opportunities to extract microscopic spatiochemical information for a broad range of alteration colors in jade artifacts.

## Introduction

Since the early Neolithic Age (9,000 BP), jade has been used to make tools, ornaments, amulets, ritual utensils, sacrificial utensils, auspicious utensils, ceremonial utensils, etc. and played a very important role during the embryonic stage of Chinese civilization [1]. Afterward, jade became a metaphor for virtue and an expression of good wishes, deeply rooted in Chinese social life. After the burial, the chemical compositions and microstructures of jade artifacts were gradually changed by weathering effect, resulting in alteration colors such as white, black, red, yellow, brown, green, and blue (called the “alteration effect”), which have been widely studied as the most prominent features of unearthed jade. In addition to the natural weathering effect, human behaviors before burial could also cause secondary color alterations [2].

The term “Shuiyin Qin (水银沁, mercury alteration)” or “Heiqigu (黑漆古, black lacquer antique)” for black alteration has been used by researchers from the late Qing Dynasty [3] to now. Previous studies showed that some black alterations contain carbon (graphite), iron, manganese, copper, and other elements [2,4–6]. However, whether mercury blackens the jade remains controversial for the lack of archaeological evidence. Until recent years, black-altered jade artifacts containing mercury were finally found in some tombs in the Pre-Qin Period (before 221 BC) (see Table S1), shedding light on a new understanding of black alteration [7–14]. Most of the abovementioned tombs contain a large number of funerary objects, indicating a higher status of the owners. Therefore, the study of blackening mechanisms may provide critical information for the conservation of cultural heritage, archaeology, and history.

A number of compounds were detected in black alteration areas, including metacinnabar (β-HgS), metallic Hg, and mercury oxide (HgO). Analyzing the burial position (whether close to the corpses) [7] and Hg/S ratio [9] indicated that the black alteration consisted of black HgS that came from the reaction between sulfur in the corpse and mercury poured into the tomb. Mai et al. [8] found that coexisting energy dispersive spectroscopy signals from S and Hg elements and characteristic micro-x-ray diffraction (μ-XRD) peak from β-HgS at the black altered areas of jade artifacts from Yuehe Tomb and inferred that the black alteration was β-HgS transformed from buried cinnabar (α-HgS). Metallic mercury alteration was ruled out according to a simulation experiment [11], indicating that pure mercury was unable to penetrate into the jade. Bao et al. [10] suggested that the black alteration may be attributed to mercury oxide when the jade artifact was heated. In addition, a loose structure with a reduction of Si, Ca, and Fe elements [8] and unoriented fibrous serpentine crystals [13] were found near the black-altered areas in jade artifacts under scanning electron microscope (SEM). Despite previous research, the form and origin of the Hg element in black alteration are still under debate. Moreover, although noninvasive imaging could be acquired by μ-XRD, SEM, and energy-dispersive spectroscopy, ultrathin layer of only a few microns near the sample surface could be extracted. In addition, the experimental methods mentioned above rely on large instruments and require invasive sample processing, limiting the applications to study precious artifacts. Therefore, rapid, effective, and nondestructive methods are preferred to reveal the blackening mechanism of jade.

Microscopy techniques based on nonlinear optics provide noninvasive 3D imaging capability, because of the fast decay of nonlinear optical signals away from the tight laser foci, and minimum thermal or mechanical disturbances to the samples [15–17]. Nowadays, nonlinear optical microscopy techniques have played important roles in the characterization of cultural heritages [18,19]. Multiphoton excited fluorescence microscopy has been used in many types of historical heritages, including varnishes [20,21], pigments [22], and metal corrosion [23]. Femtosecond pump–probe microscopy has been widely applied in chemical analysis, material science, and biology research [24–27]. In the field of archaeology, pump–probe microscopy has been applied in noninvasive study on pigments used in paintings [28–30], which is shown to be helpful in art characterization, authentication, preservation, and conservation. Second harmonic generation (SHG) is sensitive to noncentrosymmetric materials, such as cellulose, starch, polysaccharides, collagen fibers, and crystalline minerals. Hence, cultural heritages based on biological materials such as skin-based objects [31], woods [32], and starch-based adhesives [33] could be studied with SHG microscopy. Besides, multimodal nonlinear optical microscopy is capable of capturing various chemical and structural information of cultural heritages.

In this work, an ancient jade artifact of the late Spring and Autumn period (546 to 476 BC) was analyzed with combined pump–probe and SHG microscopy. Our results confirmed that the “Shuiyin Qin (水银沁, mercury alteration)” phenomenon originated not from pure mercury (Hg) but rather from β-HgS converted from α-HgS. A 3-dimensional (3D) reconstruction of the α-HgS and black alteration was further performed with a penetration depth of up to 38 μm, and the spatial relationship between α-HgS and β-HgS was analyzed, providing a high-resolution, nondestructive method for studying the alteration mechanism of ancient jade.

## Results

### Approach

**Fig. 1.**
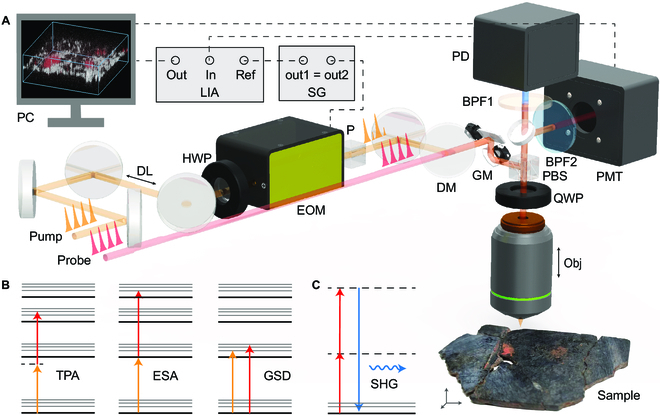
Schematic of the experimental setup. (A) Optical layout of the pump–probe/SHG microscope. Pump and probe beams first come out from the laser and then combined and scanned by 2 galvo mirrors (GMs) for laser-scan imaging. The nonlinear signals are generated and collected by photodiode (PD) or PMT after properly filtering. For pump–probe microscopy, the laser beams pass through a quarter-wave plate (QWP) before and after hitting the sample so that the polarization of the excitation and signal light could be turned perpendicular to each other, being able to be separated by a polarized beam splitter (PBS). The pump beam is intensity-modulated by EOM at a high frequency of 20 MHz and demodulated by LIA. DL, delay line; HWP, half-wave plate; P, polarizer; DM, dichroic mirror; BPF, band-pass filter; M, mirror; SG, signal generator. (B and C) Schematic of accessible nonlinear optical processes: (B) ultrafast process detected by pump–probe technique: TPA, ESA, and GSD; and (C) SHG process. Solid lines indicate discrete energy levels, the bottom solid line shows the ground state, and dashed lines indicate virtual states.

Figure 1 shows the schematic of our home-built multimodal microscopy system and nonlinear optical processes involved in our measurement. Two linearly polarized laser beams from a femtosecond optical parametric oscillator (Insight DS+, Spectra Physics) were collinearly overlapped and introduced into a laser scanning microscope system (Olympus, FV1200). In the microscope, laser beams were deflected by a pair of galvo mirrors and then passed through an objective lens (Olympus, UPLSAPO20X, numerical aperture = 0.75). Various nonlinear optical signals were generated when the tightly focused laser pulses interacted with the sample, and, in this work, all the signals were collected in backscattering geometry (epi-detection). A lateral resolution of ~0.80 μm, an axial resolution of ~7.7 μm, and a temporal resolution of ~280 fs (see Fig. S1) could be achieved. To detect the pump–probe signal, we modulated the pump beam (1,040 nm) by an electro-optical modulator (EOM; Thorlabs, EO-AM-R-20-C2) at ~20 MHz, and the differential absorption of the probe beam (~800 nm) was demodulated by a lock-in amplifier (LIA; Zurich Instruments, HF2LI). In the pump–probe process, the pump pulse excitation of molecules from ground to excited states changes the population distributions and consequently alters the absorption of the probe pulse that arrives after a certain amount of delay time, as illustrated in Fig. 1B. When the probe photon energy is close to the energy gap between the ground state and the excited state, the population decreasing in the ground state reduces the absorption of the probe beam, causing ground-state depletion (GSD). When the probe photon energy is close to the energy between 2 excited states, the population increasing in the first excited state may enhance the absorption of the probe beam, causing excited-state absorption (ESA). Moreover, the process where the molecular excitation energy requires the sum of the pump and probe photons is known as 2-photon absorption (TPA), during which the absorption of the probe beam is enhanced by the presence of the pump beam. We followed the convention of differential absorbance (−*ΔT*/*T*) signal to represent our measurements, hence the intensity loss of the probe beam (such as in TPA or ESA) appeared as positive signal; whereas the intensity gain (such as in GSD) was shown as negative signal. Pump–probe measurements reveal the transient excited-state relaxation dynamics at the femtosecond or picosecond time scales, which serve as the molecular “fingerprints” in the time domain. In addition to pump–probe signal, SHG process may also occur, with the excitation photon frequency *ω* doubled to 2*ω*, as illustrated in Fig. 1C. SHG process conserves the coherence of the excitation photons and requires the selection rule of breaking inversion symmetry.

### Pump–probe analysis of standard α-HgS, β-HgS, and metallic Hg

**Fig. 2.**
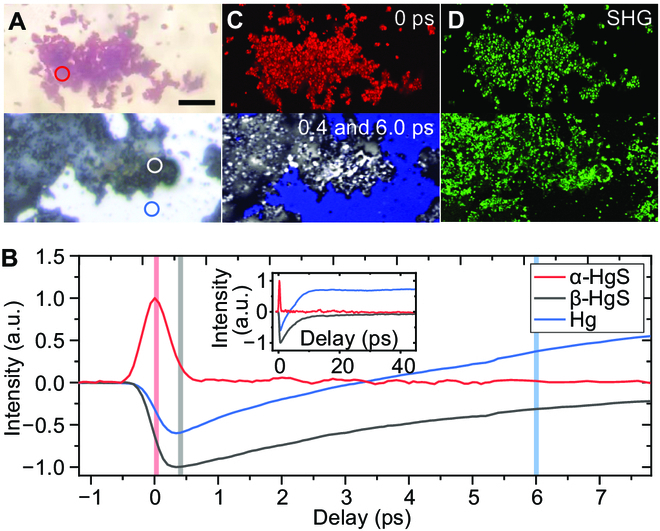
Nonlinear optical identification of standard α-HgS, β-HgS, and mercury (Hg). (A) Bright-field optical images of α-HgS (top), and mixed β-HgS and Hg (bottom). (B) Pump–probe delay profiles of the 3 standard chemicals were obtained at the position indicated by red, light gray, and blue circles in (A), respectively, with a pump/probe wavelength of 1,040/800 nm. Lighter vertical solid lines indicate the delays we chose for pump–probe imaging in (C), i.e., 0, 0.4, and 6.0 ps for α-HgS, β-HgS, and Hg, respectively. Inset: Delay profiles at a larger time scale. (C and D) False-colored images of mixed standard chemicals of the same FOV. (C) Pump–probe image. (D) SHG image. Red, blue, and gray indicate pump–probe imaging of α-HgS, β-HgS, and Hg, respectively; green indicates SHG imaging. Scale bar, 50 μm. a.u. arbitrary units.

According to previous study on this jade artifact, common elements of jade containing O, Si, Mg, and Ca were found [8], with higher concentrations of Hg and S that are associated with the secondary black material. Therefore, we first characterized the nonlinear optical properties of the 2 mercurate forms: β-HgS and powdered mercury, both of which are possible to cause the black alteration in the jade artifacts and possible to be converted from α-HgS. Therefore, standard chemicals including α-HgS (99%; Aladdin, M116112), β-HgS (prepared by liquid Hg and sulfur powder), and metallic Hg (99.999% metals basis; Aladdin, M112545) were studied as reference. The mixture of the chemicals was sandwiched between 2 coverslips, and the bright-field image showed distinct optical contrasts (Fig. 2A). Transient relaxation dynamics were measured by scanning the interpulse time delays (*τ*) between the pump and probe pulses (Fig. 2B), with the pump/probe wavelengths selected at 1,040/800 nm and laser powers of 5/5 mW on the sample. Signals were obtained in the 3 circled areas of the samples in Fig. 2A, which presented characteristic transient behaviors of the 3 chemicals. Red α-HgS appeared as a positive transient absorption signal, primarily attributed to the TPA process [30], showing the time domain “cross-correlation” line shape between the pump and probe pulses without any obvious excited-state relaxation feature. The quadratic power dependence also agreed with a TPA process (Fig. S2). Black β-HgS and Hg showed remarkable negative bleaching signals (attributed to GSD) with slow relaxation processes. The transient dynamics of liquid Hg experienced a decay of negative component and a much slower relaxation of the residual positive signal (see Fig. 2B inset), which is dominated by the ESA process. Since the decay profiles of 3 chemicals showed characteristic signatures, pump–probe microscopy could serve as a means for the specific differentiation of the 3 mercury compounds, as shown in Fig. 2C. The delay profiles at each pixel in a field of view (FOV) form a 3D image stack (*x*, *y*, *τ*). On the basis of their decay signatures, we identified the chemical compositions of α-HgS, β-HgS, and Hg by taking the signals at 0 ps (peak of positive TPA signal), 0.4 ps (valley of negative GSD signal), and 6.0 ps (positive ESA signal), respectively (detailed in Note S1). Therefore, femtosecond pump–probe microscopy has demonstrated the chemical-specific and high-resolution imaging capability to resolve the 3 mercury compounds. In addition, SHG images of the mixed samples could be taken to map α-HgS and β-HgS (Fig. 2D) because they belong to the noncentrosymmetric 152 and 216 space groups [34]. However, SHG is difficult to further differentiate the 2 forms of HgS.

Moreover, the imaging depth profile of pump–probe microscopy in jade was characterized by analyzing the full width at half maximum (FWHM) of the depth-dependent signal intensity, as shown in Fig. S3. β-HgS powder (350 μm in thickness) and liquid Hg samples were prepared, and imaging depth of 38 and 13 μm were achieved, respectively.

### Chemical-resolved nondestructive investigation of a jade artifact

We then applied the multimodal nonlinear optical imaging technique to study an ancient jade artifact from the late Spring and Autumn period, unearthed from the tomb of Yuehe, Zuozhuang Village, Yuehe Town, in Nanyang, Henan province, preserved by Nanyang Municipal Institute of Cultural Relics and Archaeology [35]. Its digital photo is shown in Fig. 1A. The jade is bluish-white tremolite with red cinnabar clay attached to the surface and penetrated black alteration. Previous archaeometrical research using x-ray photoelectron spectroscopy (XPS) [8] on jade artifacts unearthed from this very tomb was unable to determine the source of mercury in black alteration because of the overlapping and unresolvable XPS spectra. Although μ-XRD could exclude the existence of metallic mercury, the experiments required synchrotron radiation, which was inconvenient for on-site archaeological research. In addition, neither of the methods could take measurements on unprocessed samples without complex sample preparations.

**Fig. 3.**
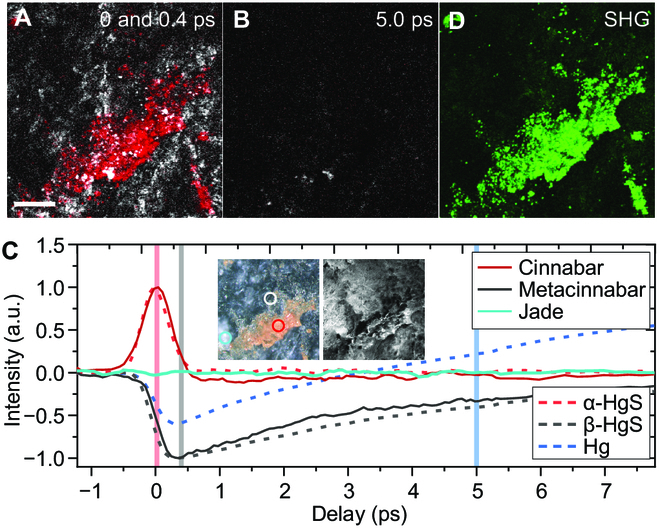
Nonlinear optical analysis of the surface of a black-altered ancient jade artifact in the Spring and Autumn period containing black alteration. (A) Pump–probe images of the jade surface. Positive TPA signal taken at 0 ps is shown in red for cinnabar, and the negative GSD signal taken at 0.4 ps is shown in white for metacinnabar. (B) Pump–probe image taken at 5.0 ps. No ESA signal from mercury was found, indicating a β-HgS-originated rather than a mercury-originated black alteration. (C) The pump–probe delay profiles of identified mercury compounds and the jade substance were obtained at the areas marked in (B). Inset: Bright-field optical image (left) and SEM image (right). Dashed lines indicate delay profiles of standard chemicals in Fig. 2. for reference. (D) SHG image. Scale bar, 50 μm.

Pump–probe imaging was performed on the jade surface and pseudo-colored (Fig. 3A and B), under the same experimental conditions as in Fig. 2. The jade surface generated intense transient absorption signals, and images taken at 0 and 0.4 ps were merged to show the distributions of TPA (red) and GSD (white), corresponding to α-HgS and β-HgS, respectively (Fig. 3A). The absence of any ESA signal (Fig. 3B) implied that the jade surface contained no detectable pure mercury, but α-HgS and β-HgS. We further examined the pump–probe decay profiles of identified α-HgS, β-HgS, and the bare jade substance from the circled areas (Fig. 3C), with the inset showing the corresponding optical and SEM images of the investigated areas. It could be seen that the transient responses of red α-HgS and black β-HgS on the surface showed consistent decay characteristics with the standard chemicals (dashed lines). In contrast, negligible transient optical signal of jade itself could be detected because of the lack of light absorption in the near-infrared window, which indicated that the analysis of mercurates was not interfered by the jade substance. According to the excavation report [35], α-HgS is widely found in tombs and coffins, and jade artifacts with black alteration were also found in the cinnabar clay layer. Our measured pump–probe optical response of the cinnabar clay obtained from the tomb matched well with that of the standard α-HgS and the cinnabar on the jade surface (Fig. S4). Combining these experimental results and the burial environment, it could be inferred that the black alteration was indeed originated from β-HgS, rather than the pure mercury (liquid Hg). The β-HgS was most likely to be transformed by the structural change from α-HgS in the tomb [35]. We also performed SHG imaging for the 2 mercurates (Fig. 3D), strong SHG signal appeared in α-HgS, well-agreeing with the distribution measured by pump–probe microscopy. The much weaker SHG response in β-HgS may result from the disruption of the regular crystal structure of β-HgS in the penetrated layer.

### 3D reconstruction of the black alteration layer

**Fig. 4.**
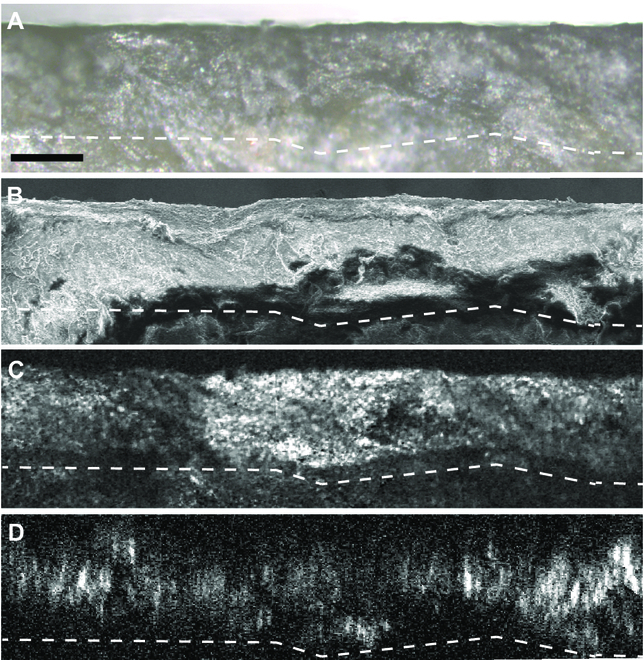
Demonstration of the 3D imaging capability. (A) Bright-field optical image and (B) SEM image of a cross-section of the jade artifact. (C) Pump–probe image taken at the same physical section. (D) Projected side-view of the pump–probe 3D image stack taken from the top surface. Little signal from α-HgS was shown because almost no α-HgS had penetrated the jade. Scale bar, 50 μm. White dashed line indicates visual guide.

One key advantage of nonlinear optical microscopy is the noninvasive 3D imaging capability, which could be hardly achieved by traditional surface science analytical approaches such as XPS and XRD. The intrinsic optical sectioning ability resulted from the nonlinear relationship between optical signal and excitation laser intensity, which is essential to access the 3D spatial distribution and the formation process of alteration color inside the unearthed jade artifacts. Here, we demonstrate nondestructive 3D analysis on a small fresh section of a jade fragment, with its optical, SEM, and pump–probe images of the same FOV shown in Fig. 4. The white dashed line served as a visual marker for the black alteration layer, and β-HgS enrichment was shown to appear in about 30 μm from the surface. Heavier Hg element in the alteration color appeared brighter in the SEM image near the surface (Fig. 4B). Pump–probe images were extracted at 0.4 ps to represent β-HgS that penetrated into the jade. The pump–probe image acquired directly at the physical cross-section of the fragment (Fig. 4C) has an overall agreement with the corresponding optical and the SEM images. A similar image can also be projected from the depth-scanned 3D image stack taken from the top surface of the jade fragment (Fig. 4D). The agreement between the physical section (Fig. 4C) and the reconstructed nonlinear optical section (Fig. 4D) verified a nondestructive method with sufficient penetration depth. In addition, through volumetric imaging, a section in any direction could be generated, showing the distributions of α-HgS or β-HgS related to irregular inner structures of the jade.

**Fig. 5.**
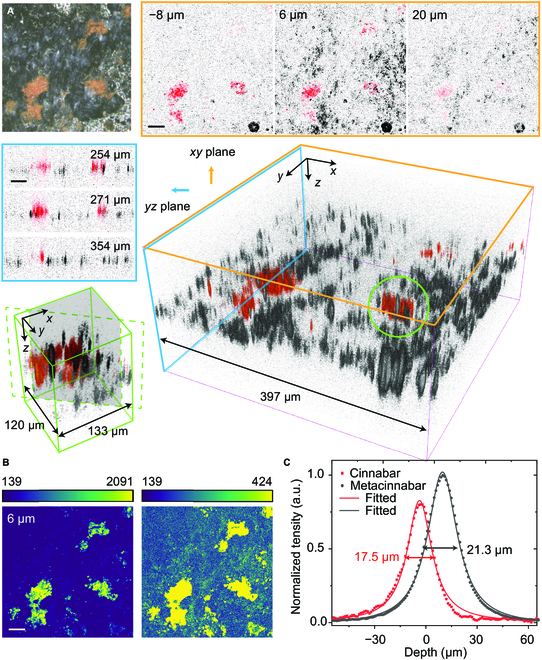
Nondestructive 3D analysis of the black alteration in the jade artifact. (A) 3D reconstruction of pump–probe images taken from the α-HgS (red) attached onto the surface and the black β-HgS (black) penetrated into the jade. Top left: Bright-field optical image. Nonlinear optical cross-sections from *xy* (orange box) and *yz* (blue box) planes are shown in top and left panels. Bottom left: zoom-in view of the area indicated by the green circle with a virtual cross-section. Scale bar, 50 μm. (B) SHG images of α-HgS and β-HgS of a same FOV with different calibration bars. (C) Normalized overall depth profiles of α-HgS and β-HgS. The distribution of α-HgS appears at a shallower depth with a narrower range than β-HgS.

Detailed 3D reconstruction and analysis of the black alteration inside the ancient jade artifact are shown in Fig. 5. Optical images of the sample were taken before and after nonlinear optical measurements, confirming no photo damage had occurred. While optical image did not provide the depth profile of the colored α-HgS and β-HgS, pump–probe microscopy clearly revealed their 3D distributions in the unearthed jade artifact, with a volume of ~ 400 μm × 400 μm × 190 μm. Since the jade sample appeared to contain no metallic Hg, we chose the penetration of β-HgS, that is, 38 μm as the overall penetration depth. A larger depth of 190 μm was scanned for a complete 3D view. Multiple views of the cross-sections at the *xy* and *yz* planes at different positions were extracted and showed the variation of the 2 chemical distributions along different directions (Fig. 5A). Our results showed that α-HgS existed within a shallower layer than β-HgS (bottom panel of Fig. 5A), indicating that α-HgS was mainly attached to the surface of the jade, while the black β-HgS penetrated deeper into the interior of the jade. ESA signal generated by mercury was still undetectable throughout the whole imaging volume. Figure 5B shows the 3D reconstruction from the SHG (green) of the same FOV where α-HgS dominated the signal and agrees with the pump–probe results. Lower SHG intensity at the black alternated areas may indicate smaller microcrystal sizes of β-HgS, which may be easier to penetrate into the jade and form the black alteration. Figure 5C shows the z-profile of pump–probe signal intensities integrated within the *xy* planes and their Voigt fits (solid lines). The intensities were normalized to the maximum signal of β-HgS. The FWHM and peak position of the 2 depth profiles suggest the relative distributions of the 2 chemicals: The overall existence of β-HgS was ~3.5 μm deeper than α-HgS. Moreover, the larger FWHM of β-HgS and the spatial overlap between the 2 mercurates support that black β-HgS alteration may be transformed from attached α-HgS.

## Discussion

The degradation of α-HgS (vermilion) has been a topic for researchers because α-HgS has a long history of used as red pigment in many kinds of artworks, such as oil paintings, tempera, or mural, in many countries. β-HgS and metallic mercury have also been discussed as the possible reaction products. In the case of black alteration in the jade artifact from Yuehe Tomb, β-HgS led to the darkening. It is of great significance to discuss the alteration mechanism. Dreyer [36] suggested that alkali (KOH in his experiment) promotes α-HgS darkening. Halides such as chloride were widely considered as a reason for α-HgS to β-HgS transformation as catalytic elements [37–40]. According to Elert et al. [41], sulfate may offer another pathway for α-HgS darkening through forming soluble mercury sulfate hydrate (HgSO_4_·H_2_O) at high relative humidity. The Yuehe Tomb was discovered during the exploration of trona mineral, which suggests that the soil near the jade in the tomb may be alkaline and contain sulfate and chloride. Therefore, it may be inferred that the α-HgS in the tomb transferred to small even amorphous β-HgS [37,42] with the help of water, alkali, chloride, and sulfate. β-HgS penetrated into the jade subsequently, forming the black alteration.

Comparison of the depth profile of α-HgS and β-HgS near the surface reveals a lamination of these 2 materials, emphasizing that the β-HgS was likely to be transformed from the α-HgS. In addition, metallic mercury shows characteristic ESA signal with a distinct decay profile, allowing accurate and convenient optical analysis on jade samples. In contrast, metallic mercury studied by electrochemistry was not directly applicable by XRD methods [42]. Our results unambiguously excluded the existence of metallic mercury in Hg compounds by pump–probe microscopy. The major limitation of the current technique is the imaging depth, which could be improved with optimized index matching liquids for the microscope objectives, or using advanced depth imaging methods, such as adaptive optics [43]. Increasing integration time, implementing line- or frame-averaging would also improve the image quality, while keeping relatively low laser excitation power to avoid sample damage or photobleaching. Our imaging method requires only a few minutes to obtain the 3D reconstruction of a compound in a typical volume of ~ 400 μm × 400 μm × 190 μm in our setup. Combined with the motorized translation stage for mosaic stitching, large-area imaging could be achieved [44–46], enabling the possibility to analyze the whole vessel of large jade artifacts. On the other hand, the development of high-stability laser sources and miniaturized probes for nonlinear optical imaging will also help the on-site and in situ researches at archaeological sites.

### Conclusions

In conclusion, our results demonstrated the capability of combined pump–probe and SHG microscopy for nondestructive 3D analysis of jade artifacts. In unearthed jade artifacts from the late Spring and Autumn period, we have performed chemical-specific imaging of the black alteration, confirming that the “mercury alteration” in ancient jade was originated from β-HgS rather than pure mercury (Hg). Our technique may be extended to find out the influence of burial time, burial environment, and jade quality on the alteration colors. A database based on the pump–probe features of alteration colors with different components may also be built to give a systematic understanding of alteration effects in jade artifacts.

## Materials and Methods

### Nonlinear optical microscopy

Linearly polarized pump and probe beams emitted from a femtosecond optical parametric oscillator (Insight DS+, Spectra Physics) at a repetition rate of 80 MHz of ~200 fs. The 2 pulses were combined and introduced into a laser scanning microscope system (Olympus, FV1200) and focused by an objective lens (numerical aperture = 0.75). Signals were detected in backscattering geometry. For pump–probe microscopy, pump pulses were intensity-modulated by an EOM (Thorlabs, EO-AM-R-20-C2), and the modulation on the pump beam was transferred to the probe beam after the 2 beams interacted with the sample successively. Two beams were combined by a dichroic mirror, the time delay between which was adjusted by an optical delay line driven by a motorized stage (Newport, controlled by Model ESP301). The probe beam at different time delays (*τ*) was demodulated by an LIA (Zurich Instruments, HF2LI) and collected by silicon photodiode after a bandpass filter (Chroma, ET890/220M). The laser beams passed through a quarter-wave plate (Thorlabs, AQWP05M-980) before and after hitting the sample so that the polarization of the signal light was 90° rotated with respect to the excitation light, making it possible for them to be separated by a polarized beam splitter (Thorlabs, PBS103). For SHG microscopy, signal was collected by photomultiplier tubes (PMTs) after a bandpass filter (Semrock, FF01-405/10-25). Because of the high signal-to-noise ratio provided by EOM and LIA, the pixel dwell time of the pump–probe microscopy can be as low as 2 μs, achieving a framerate of about 1 frame/s with a FOV of 400 μm × 400 μm. SHG signal can also be collected at the same rate by PMT, providing a quick method with chemical and structural resolution for analyzing jade alteration.

### Scanning electron microscopy

The surface morphology of a section of the jade was characterized by Zeiss Sigma 300 (Carl Zeiss AG). The images were obtained in high vacuum mode (5.07 × 10^−7^ torr) at the acceleration voltage of 5 kV under 30-mm aperture and a working distance of 7.1 mm using the second electron detector SE2.

### Standard chemical preparation

Liquid Hg (99.999% metals basis; Aladdin, M112545) and sulfur powder (99.9%; Liangfeng) were 1:1 mixed to produce β-HgS. We waited for a week to ensure that the chemical reaction was fully proceeded. The produced β-HgS, liquid Hg, and standard α-HgS (99%, Aladdin; M116112) were put between 2 cover glasses for further characterization.

### Nondestructive investigation of intact jade artifact

The jade artifact was unearthed from the tomb of Yuehe, Zuozhuang Village, Yuehe Town, in Nanyang, Henan province, preserved by Nanyang Municipal Institute of Cultural Relics and Archaeology. According to the inscriptions on a cooper bell, the tomb belongs to Shou, a king of Yang State in the late Spring and Autumn period. The artifact was bluish white with a large black alteration, and some adhered red spots. To demonstrate the 3D imaging capability of our method, a small sample was cut from a fragment of the same artifact for the cross-sectional imaging.

### Image data processing and 3D reconstruction

We processed the image data stacks by the open-source software ImageJ [47] and reconstruct the jade artifact surface by the open-source software 3D Slicer.

## Data Availability

The data could be given upon reasonable request form the corresponding authors.

## Supplementary Materials

Supplementary 1 Note S1 Fig. S1. System characterization. Fig. S2. Power dependence of pump–probe signals of the 3 standard chemicals indicating the nonlinear origin of pump–probe signal in standard chemicals. Fig. S3. Depth dependence of pump–probe intensity. Fig. S4. Characterization of the unearthed cinnabar clay. Table S1. Tombs where the unearthed jade artifacts with black alteration contain mercury.
