# Supplementary material for: Nondestructive Nonlinear Optical Microscopy Revealed the Blackening Mechanism of Ancient Chinese Jades
Source: Research (Wash D C). 2023 Nov 14;2023:0266. doi: 10.34133/research.0266 (PMC10644832; doi:10.34133/research.0266)
Supplement: Supplementary 1 — Note S1 Fig. S1. System characterization. Fig. S2. Power dependence of pump–probe signals of the 3 standard chemicals indicating the nonlinear origin of pump–probe signal in standard chemicals. Fig. S3. Depth dependence of pump–probe intensity. Fig. S4. Characterization of the unearthed cinnabar clay. Table S1. Tombs where the unearthed jade artifacts with black alteration contain mercury. [file research.0266.f1.docx]

Supplementary Materials

Note S1

Fig. S1. System characterization.

Fig. S2. Power dependence of pump-probe signals of the three standard chemicals indicating the nonlinear origin of pump-probe signal in standard chemicals.

Fig. S3. Depth dependence of pump-probe intensity.

Fig. S4. Characterization of the unearthed cinnabar clay.

Table S1. Tombs where the unearthed jade artifacts with black alteration contain mercury.

**Note S1**

**Decomposition of the Chemicals from the Mixed Ultrafast Delay Profile**

Firstly, for only pure Hg contributes to the ESA signal, the existence of pure Hg could be confirmed from the positive signal at ~ 6 ps. Then, for pure both Hg and β-HgS contribute to the negative GSD signal, we extracted β-HgS image by subtracting the Hg image taken at ~ 6 ps from the image taken at ~ 0.4 ps. Finally, positive TPA signal at ~ 0 ps indicated α-HgS, weak negative GSD signal from β-HgS or pure Hg was removed. By merging the 3 channels, we get false-colored image indicating the spatial distribution and relative content of the mercurates.


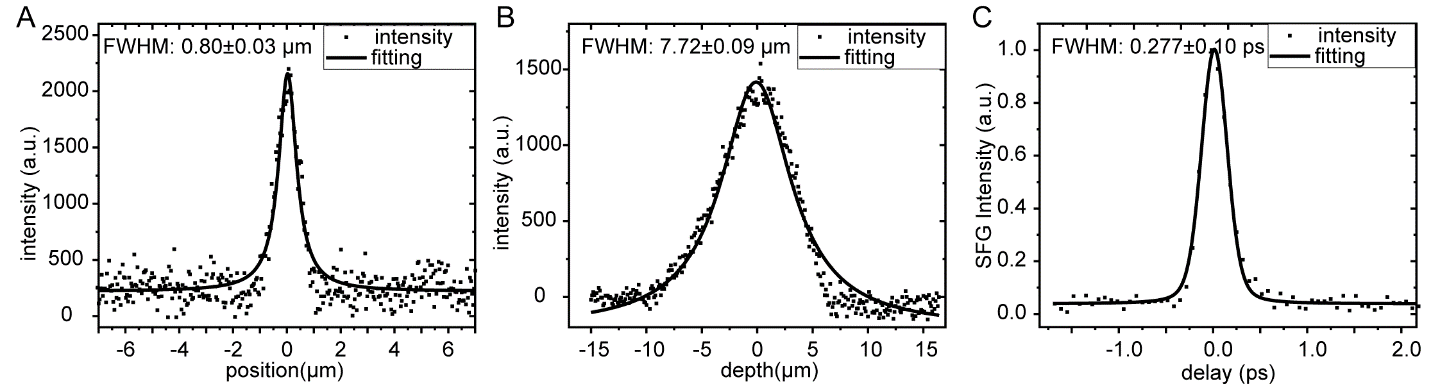


**Fig. S1. System characterization.** (A) Lateral resolution; (B) depth resolution and (C) temporal resolution of the setup.


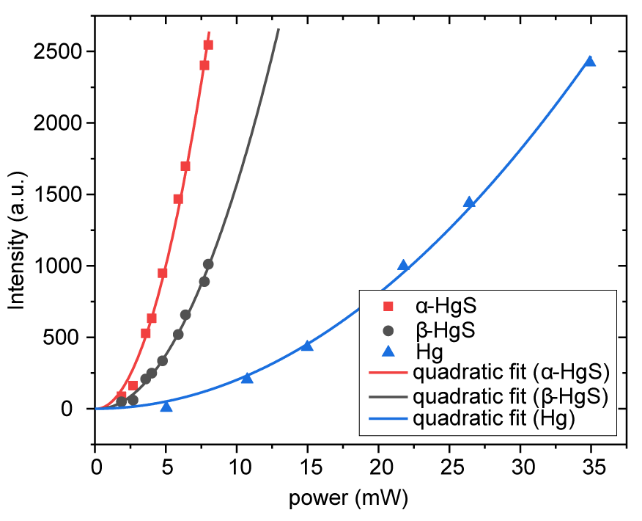


**Fig. S2. Power dependence of pump-probe signals of the three standard chemicals indicating the nonlinear origin of pump-probe signal in standard chemicals.**


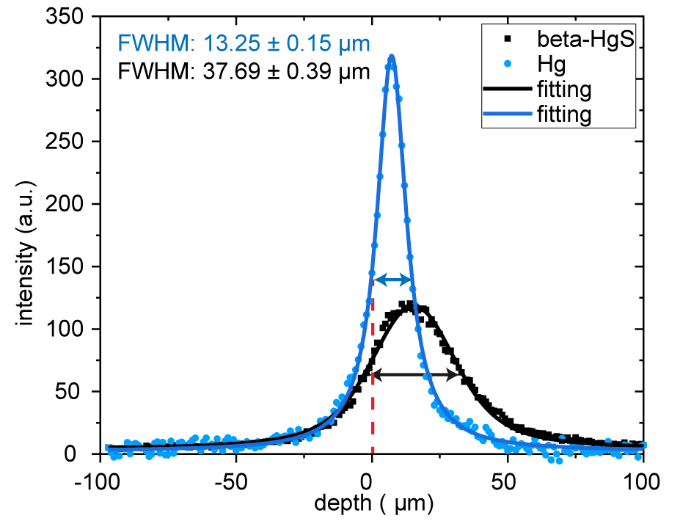


**Fig. S3. Depth dependence of pump-probe intensity.** Red dashed line shows the z coordinate corresponding to half of the maximum intensity.


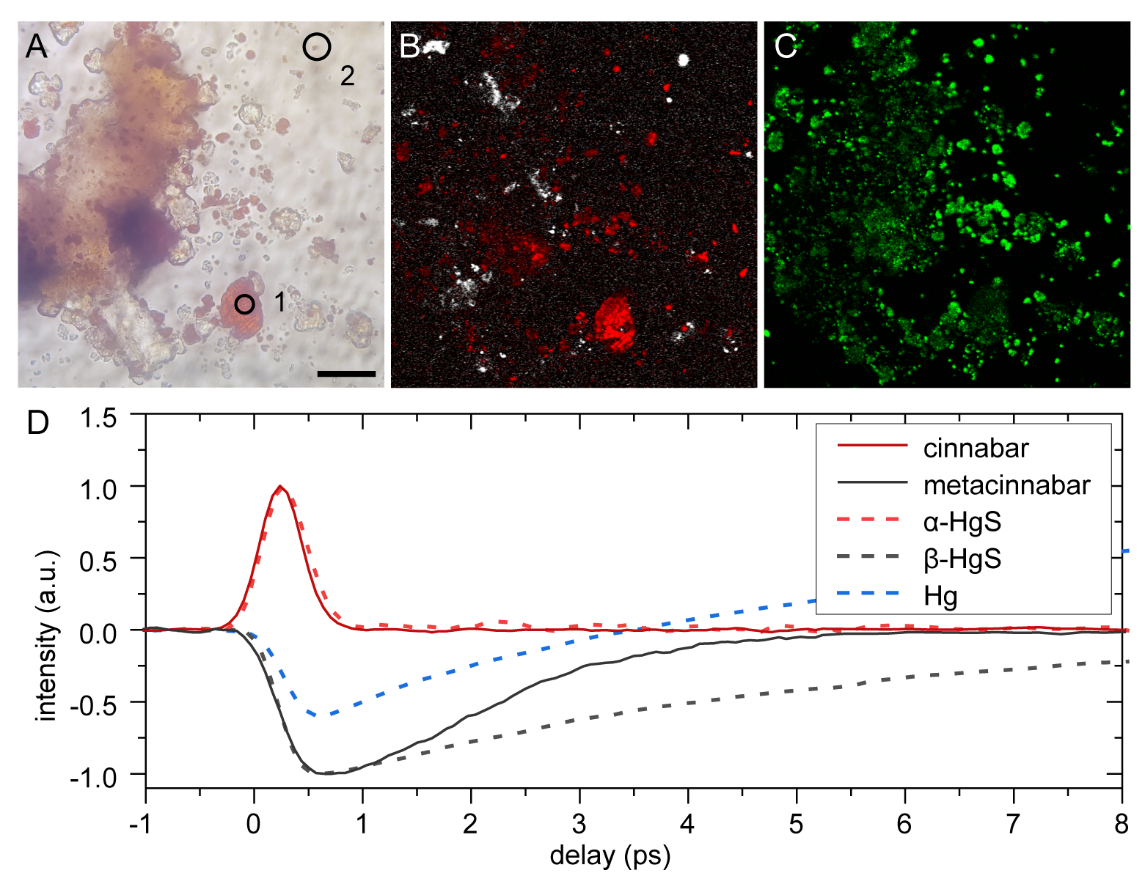


**Fig. S4. Characterization of the unearthed cinnabar clay.** (A) Bright-field optical image. (B) pump-probe (in red and white), (C) SHG (in green) image. (D) Pump-probe delay profiles of different positions indicated in (a).

**Table S1. Tombs where the unearthed jade artifacts with black alteration contain mercury.**

| Location | Tomb | Excavation | Tomb Grade | Time | Method for Studying Hg | References |
| --- | --- | --- | --- | --- | --- | --- |
| Anyang, Henan Province | Yinxu (Ruins of Yin) | ~ 1930s-1980s | High | Late Shang Dynasty (1300-1046 BC) | XRF | Wang et al.^14^ |
| Nanyang, Henan Province | Yuehe Tomb No.1 | 1993-1994 2001-2002 | High | Late Spring and Autumn period (546-476 BC) | XRF | Xu et al.^7^ |
|  |  |  |  |  | XRF, SEM-EDS, XPS, μ-XRD | Mai et al.^8^ |
|  |  |  |  |  | XRF | Chen et al.^9^ |
| Jing’an, Jiangxi Province | Lizhou’ao Tomb | 2007 | High / middle | Late Spring and Autumn period (546-476 BC) | SEM-EDS, CT | Bao et al.^10^ |
| Changsha, Hunan Province | Yangjiashan, Tomb No. 12 | 1960 | Middle | Warring States period (284-221 BC) | XRF, simulation experiment | Zhao et al.^11,12^ |
| Changde, Hunan Province | Jiuli Tea Factory Tomb No. 1 | 1980 | High | Middle Warring States period (386-285 BC) |  |  |
